# Framing the ultimatum game: the contribution of simulation

**DOI:** 10.3389/fnhum.2013.00337

**Published:** 2013-07-09

**Authors:** Barbara Tomasino, Lorella Lotto, Michela Sarlo, Claudia Civai, Rino Rumiati, Raffaella I. Rumiati

**Affiliations:** ^1^IRCCS E. Medea-Associazione La Nostra FamigliaSan Vito al Tagliamento, Italy; ^2^Dipartimento di Psicologia dello Sviluppo e della Socializzazione, Università di PadovaPadova, Italy; ^3^Dipartimento di Psicologia Generale, Università di PadovaPadova, Italy; ^4^Cognitive Neuroscience Sector, SISSATrieste, Italy

**Keywords:** ultimatum game, framing effect, anterior insula

## Abstract

It has now become widely accepted that economic decisions are influenced by cognitive and emotional processes. In the present study, we aimed at disentangling the neural mechanisms associated with the way in which the information is formulated, i.e., framing effect, in terms of gain or loss, which influences people's decisions. Participants played a fMRI version of the Ultimatum Game (UG) where we manipulated bids through two different frames: the expression “I give you” (*gain*) focusing on money the respondent would receive if she/he agreed with the proponent, and the expression “I take” (*loss*) focusing on the money that would be removed from the respondent in the event that she/he accepted the offer. Neuroimaging data revealed a frame by response interaction, showing an increase of neural activity in the right rolandic operculum/insular cortex, the anterior cingulate, among other regions, for accepting the frame “I take” vs. rejecting, as compared to accepting the frame “I give you” vs. rejecting. In addition, the left occipito-temporal junction was activated for “I take” vs. “I give you” for offer 5, corresponding to the equal offer made unpleasant by the presence of the frame “I take,” where is the proposer that takes the money. Our data extend the current understanding of the neural substrates of social decision making, by disentangling the structures sensitive to the way in which the information is formulated (i.e., framing effect), in terms of gain or loss.

## Introduction

Cross-field research in experimental economics and cognitive psychology has clearly demonstrated how both the cognitive and emotional processes may influence economical decision-making (Bechara et al., 1997; Sanfey et al., 2003; Naqvi et al., 2006). More critically, these studies unveiled the limits of the theory of rationality proposed by von Neumann and Morgenstern (1947).

In the Ultimatum Game (UG), two players are asked to divide a given amount of money: the proponent must decide how this money should be divided, while the responder may accept or reject the offer. If the responder accepts the offer, both players receive the agreed amount, but if the responder rejects, neither of them gets anything. What has been observed is that when participants play as responders, they tend to reject about 5 of bids below the 2–3 of the total (Henrich et al., 2001), behaving against the predictions of classical economic theories of monetary maximization of utility (von Neumann and Morgenstern, 1947), as even the acceptance of an offer would constitute a minimal gain, and, as such, worth being accepted. The UG violates the classic assumption of the *homo economicus*, in that people prefer to reject a sure amount of money rather than accepting an unfair division. In order to explain this behavior, behavioral economists developed the concept of social preference, defined as a concern for the payoffs of other relevant agents in addition to the concern for one's own payoff (Carpenter, 2008), and proposed different accounts. According to one account, focused on the distribution of the outcomes, the individuals reject unequal offers because they have a preference for equal outcome (e.g., Bolton, 1991; Fehr and Schmidt, 1999). The second account focuses on intentions, and claims that people reject unfair offers in order to punish the socially unacceptable behavior of the proposer. From a psychological point of view, negative emotions, such as frustration, have been proposed as being the ultimate cause of the rejections (Pillutla and Murnighan, 1996), and psychophysiological, imaging and neuropsychological evidence supports this interpretation.

Van't Wout and colleagues, for instance, found that increased skin conductance response, a measure of emotional arousal, was associated with the rejection, compared to acceptance, of unfair offers (van't Wout et al., 2006). Sanfey et al. (2003), on the other hand, interpreted the stronger activation of anterior insula associated with rejection of unfairness as a sign of emotional arousal, as this area had traditionally been linked to negative emotions such as disgust (Sanfey et al., 2003). Koenigs and Tranel (2007) found that patients with a lesion to the vmPFC increased their rate of rejections for unfair offers, interpreting this result as a sign of a deficit in controlling frustration (Koenigs and Tranel, 2007). Interestingly, Moretti et al. (2009) confirmed that a lesion in the vmPFC led to an increased rate of rejection of unfair offers, but only when they were presented as future abstract outcomes; instead, when money was physically present during the interaction, their rate were no different from the control group (Moretti et al., 2009). This latter finding suggests that the vmPFC is involved in representing the value of future abstract rewards rather than in controlling negative emotions elicited by unfairness. Further evidence in support to the involvement of mechanisms other than negative emotional arousal is provided by Civai et al. (2010), who found that participants were more aroused when they rejected, as opposed to when they accepted, unfair offers for themselves: when asked to decide on behalf of an unknown third party, subjects rejected the same amount of unfair offers, but they showed no difference in the emotional arousal between rejection and acceptances (Civai et al., 2010). It could be possible that the increased arousal in myself condition is driven by the fact that subjects incur in the cost of rejecting, whereas in the third-party condition they do not; however, more recent behavioral data showed that, in other situations, the unfairness of the division does not prevent acceptance when the offer is advantageous for the responder (Civai et al., 2012, 2013), suggesting that other mechanisms besides pure perception of unfairness may drive the behavior in myself condition (e.g., willing to be better off the other player). By applying this same self-other manipulation, Corradi-Dell'Acqua et al. (2012) found that the medial prefrontal cortex was associated to rejections in the self condition, whereas the anterior insula was associated with rejection in third party condition (Corradi-Dell'Acqua et al., 2012), and suggested that the activation in anterior insula is triggered by social norm violation (e.g., King-Casas et al., 2008) and not just by emotional arousal [for a discussion on this issue, see Civai (2013), in this special issue].

In the domain of economics, a number of studies demonstrated that decision-making is strongly affected by gain and loss contexts (Tversky and Kahneman, 1981; De Martino et al., 2006). Specifically, in a modified version of the UG, participants' responses were compared in gain and loss sharing (Zhou and Wu, 2011). In the gain condition, the standard rules of the UG were applied. In the loss condition, accepting the offer led to the proposed division of the loss between players, whereas rejecting the offer led both players to lose the total amount of money. Results showed that the rejection rates to unfair offers were higher in the loss than in the gain condition. Other studies have demonstrated that the perception of ownership of property affects the way proposers make offers in the UG. In particular, proposers allocated more chips to the responder in the taking (i.e., the property is located at the responder, and the proposer decides how many chips to take from the responder) than in the giving condition (i.e., the property is located at the proposer, and the responders decides how many chips to give to the responder) (Leliveld et al., 2008). Indeed, the way in which the information is formulated, in terms of gain or loss, has been found to influence people's decisions. This effect is known in literature as the framing effect, which is one of the psychological phenomena explained within the prospect theory framework (Kahneman and Tversky, 1979), whereby people: (a) perceive the different options in terms of potential gains or potential losses compared with a neutral reference point, (b) consider the losses most salient than the corresponding gains (the unpleasantness of losing Euro 1000 is a stronger feeling than the pleasantness of winning the same amount), and (c) are more inclined to make risky choices in the domain of losses.

In the classic UG, offers are typically formulated so as to provide the respondent with all the information on how the money will be distributed between the two players: for example, if the sum to be divided is 10 euros, the offer is worded as “I take 8 Euro/You take 2 Euro” (Sanfey et al., 2003; van't Wout et al., 2006; Moretti et al., 2009). However, the two pieces of information are complementary. Therefore, on the one hand, one piece of information might be sufficient to make a decision, on the other, the way the offer is framed might well affect decision-making.

In a previous psychophysiological study (Sarlo et al., 2012), some of us used a modified version of the UG in which bids were manipulated through two different frames: the expression “I give you” was considered as a *gain* frame, since it focuses on money the respondent would receive if she agreed with the proponent; on the contrary, the expression “I take” was considered to frame the *losses*, since it is focused on the money that would be removed from the respondent in the event that she accepted the offer. Heart rate and skin conductance were also recorded in response to offers as indices of physiological activation. The results indicated that manipulating the frame had an effect both at the behavioral and physiological levels in males only. They showed a psychophysiological pattern suggesting a defense response (increased heart rate and skin conductance) when the offer was framed as a loss rather than as a gain, and a higher rate of rejection under the loss than the gain frame with mid-value offers (3 out of 10€). Accordingly, in the present study we hypothesized that the frame “I take” might elicit stronger bodily responses because it might be interpreted more negatively.

The framing effect has been investigated in two previous neuroimaging studies using a financial decision-making task (De Martino et al., 2006; Roiser et al., 2009). Consistent with the prospect theory assumptions, participants preferred the sure over the gamble options in the gain frame condition, and chose the gamble over the sure options in the loss frame condition. fMRI data showed that choices consistent with such framing effect were associated with amygdala activity, likely reflecting automatic emotional reactions (but see also Talmi et al., 2010). Other studies have indicated that risk aversion may also be mediated by activation of the anterior insula (Kuhnen and Knutson, 2005; Liu et al., 2007), suggesting that enhanced sensitivity to loss-framed information is associated with negative emotions and reward-related processing (Phan et al., 2002).

This is the first study to date that has investigated the neural mechanisms underlying framing effect in the UG. Based on these extant literature, first we expected to replicate the findings that correlate the activation of areas previously associated with unfairness, such as medial prefrontal cortex and anterior insula, to the type of response; moreover, we predicted a significant effect of loss (“I take”) vs. gain (“I give you”) frame in emotional areas such as the amygdala and anterior insula.

## Methods

### Participants

A total of 17 males right-handed [mean ± SD: 93.5 ± 9.9, Edinburgh Inventory test, (Oldfield, 1971)] healthy subjects (mean age ± SD: 27.35 ± 3.88 years; age range: 22–36) were included. Male subjects were preferred to female subjects because in Sarlo et al. (2012) they more consistently showed the effect of frame. All subjects were native speakers of Italian with comparable levels of education. All subjects had normal or corrected-to-normal vision and reported no history of neurological illness, psychiatric disease, or drug abuse according to their responses on self-report measures. None had any previous knowledge of the UG. All participants gave informed consent to participate in the study. The study was approved by the local ethics committee.

### Task and stimuli

Task, stimuli, and experimental set-up were similar to those employed in a previous psychophysiological study (for details see Sarlo et al., 2012). Participants underwent a session of 16 min. The experimental instructions (see Appendix for an English-translated instruction sheet) can be subsumed as follows: participants played as *responders*. They were told that previous participants played as *proposers* and made offers by deciding how to split the amount of 10 euro at each trial (*N* = 62) that had been available by the experimenter. The participant had to decide either to accept or to reject the offer, by pressing one of two response keys. If participants accepted the offer, both (*proposer* and *responder*) will get the money as suggested, whereas if they rejected the offer, none of the players would get any of the money.

Although participants were told that they were interacting with human proposers, they were actually presented with offers defined a priori by the experimenter. There were three possible offers (factor OFFER): unfair [1€], middle [3€], and fair [5€] (in “1€ out of 10” the *responder* is offered only 1 of the money at stake). Each offer was framed in two different ways (factor FRAME: “I give you/I take”). Each participant received the full range of offers, which were presented in different orders across subjects, with the constraints that (a) all the three offers should be presented first, and then repeated, (b) no more than two offers formulated with the same frame should appear consecutively, (c) the same amount of money (in the two different frames) should not be offered consecutively.

Participants were told that the proposers would receive feedback only at the end of the experiment (i.e., “covered” UG, which prevents strategic use of rejections; see Oldfield, 1971; Zamir, 2001; Civai et al., 2012; Corradi-Dell'Acqua et al., 2012). At the beginning of the experiment, participants were also told that their compensation for participating in the experiment would be proportional to the amount of money gained during the UG. Instead, irrespective of the task performance, they received the same amount of money as compensation after completion of the experiment. The subjects were not informed at the end of the experiment that we used a flat rate. An informal debriefing was carried out to assess whether participants believed whether offers were genuinely human.

### Experimental set-up

Participants lay supine in the MR scanner with their head fixated by firm foam pads. Presentation of the stimuli and their synchronization with the MR scanner were realized by the software Presentation® (version 9.9, Neurobehavioral Systems Inc., CA, USA). Stimuli were projected through a VisuaStim Goggles system (Resonance Technology). Subjects responded by pressing the corresponding keys of an MRI-compatible response device (Lumitouch, Lightwave Medical Industries, Coldswitch Technologies, Richmond, CA).

For each experimental trial a fixation point (500 ms) was presented, followed by the offer (e.g., “I give you/I take 5€”, 6000 ms), after which a 2 s display indicated that the response (“accept”/”reject”) could be made (“decision slide”). Trials were intermixed by inter-trial intervals ranging randomly from 3060 to 6720 ms with an incremental step of 60 ms. Instructions emphasized that the participants should press the selected key when the decision slide appeared on the screen.

Each experimental session included 62 randomized trials, including 54 experimental trials [3 (GAIN: 1€, 3€, 5€) × 2 (FRAME: “I take,” “I give you”) × 9 repetitions], yielding a total of 27 offers for each frame condition and 8 trials of no interest (2 offers with gain 2€ and 2 offers with gain 4€), were included for each frame condition in order to represent the full range of offers the hypothetical proposers would make, while keeping reasonable the total number of trials (cf. Sarlo et al., 2012). Therefore, we focused on the trials representing the very unfair, the mid-value, and the very fair offers, according to previous studies (Polezzi et al., 2008; Civai et al., 2010). Eight null events (i.e., blank screens), perceived as a prolongation of the inter-trial period, were randomly interspersed among the event trials to increase the power of estimating the BOLD response (Dale and Buckner, 1997) and 30 s. of low level baseline (i.e., fixating a cross placed at the centre of the screen for 15 s at the beginning and 15 s at the end of the experiment). We therefore investigated the effect of two factors, GAIN and FRAME. Prior to the experiment, subjects practised the task outside the scanner (*N* = 20 trials): subjects were told that, in order to acquire familiarity with the structure of the task, they had to play some fake trials on a computer outside the scanner, being informed that the offers were not real, and the subject was told that they wouldn't have been calculated in the final payoff. The offers could take any amount.

### fMRI data acquisition

A 3.0-T Philips Achieva (Philips Medical System, Netherlands) whole-body scanner was used to acquire T1-weighted anatomical images and functional images using a SENSE-Head-8 channel head coil and a custom-built head restrainer to minimize head movements. Functional images were obtained using a T2^*^-weighted echo-planar image (EPI) sequence of the whole brain. EPI volumes for the main experiment (*N* = 455, lasting 14.2 min) contained 30 transverse axial slices (repetition time, *TR* = 2000 ms; echo time, *TE* = 35 ms, field of view, *FOV* = 23 cm, acquisition matrix: 128 × 128, slice thickness: 3 mm with no gaps, 90° flip angle, voxel size: 1.79 × 1.79 × 3 mm) and were preceded by 5 dummy scans that allowed the MR signal to reach a steady state. After functional neuroimaging, high-resolution anatomical images were acquired using a T1-weighted 3-D magnetization-prepared, rapid acquisition gradient fast filed echo (T1W 3D TFE SENSE) pulse sequence (*TR* = 8.2 ms, *TE* = 3.76 ms, *FOV* = 24 cm, 190 transverse axial slices of 1 mm thickness, 8° flip angle, voxel size: 1 × 1 × 1 mm) lasting 8.8 min.

### fMRI data processing and whole brain analysis

fMRI data pre-processing and statistical analysis were performed on UNIX workstations (Ubuntu 8.04 LTS, i386, http://www.ubuntu.com/) using MATLAB r2007b (The Mathworks Inc., Natick, MA/USA) and SPM5 (Statistical Parametric Mapping software, SPM; Wellcome Department of Imaging Neuroscience, London, UK). Dummy scans were discarded before further image processing. Preprocessing included spatial realignment of the images to the reference volume of the time series, segmentation producing the parameter file used for normalization of functional data to a standard EPI template of the Montreal Neurological Institute template provided by SPM5, re-sampling to a voxel size of 2 × 2 × 2 mm, and spatial smoothing with a 6-mm FWHM Gaussian kernel to meet the statistical requirements of the General Linear Model and to compensate for residual macro-anatomical variations across subjects. We performed a whole brain random effects analysis closely following the model previously used by some of us (Civai et al., 2012), with FRAME and the RESPONSE TYPE (i.e., reject or accept) as factors to account for neural activations related to accepting or making a rejection for offers proposed as different frames. This analysis counted 13/17 subjects, because it was necessary that all the subjects considered had rejections in both the frames in order to perform an ANOVA without empty cells. We calculated the number of cells for each condition. There was a mean of 16,56 ± 9,15, 12,17 ± 8,67, 15,06 ± 9,82, and 13,28 ± 9,39 cells for each of the four conditions: I TAKE_ACCEPT; I TAKE_REJECT; I GIVE YOU_ACCEPT; I GIVE YOU_REJECT, respectively. Importantly, the mean number of cells did not differ significantly across experimental conditions [frame, *F*_(1, 12)_ = 3.37, *p* = 0.089, n.s.; resp type, *F*_(1, 12)_ = 1.012, *p* = 0.33, n.s.; frame × resp type interaction, *F*_(1, 12)_ = 0.01, *p* = 0.90, n.s.], thus cells were comparable between conditions. On the first-level analysis, we modeled as the regressors of main interest the response types (accept/reject) and the frames “I take” and “I give you” (I_take/accept, I_take/reject, I_give_you/accept, and I_give_you/reject) and their temporal derivative. We also included the motor response as a further regressor of no interest In addition, to correct for motion artifacts, subject-specific realignment parameters were modeled as covariates of no interest. Low-frequency signal drifts were filtered using a cut-off period of 128 s. At the single subject level, specific effects were assessed by applying appropriate linear contrasts to the parameter estimates of the experimental conditions resulting in *t*-statistics for each voxel. For the second-level random effects analyses, contrast images obtained from individual participants were entered into a one-sample *t*-test to create a SPM{T}, indicative of significant activations specific for this contrast at the group level. We used a threshold of *p* < 0.05, corrected for multiple comparisons at the cluster level [using family-wise error (FWE)], with a height threshold at the voxel level of *p* < 0.001, uncorrected. The anatomical localization of the functional imaging results was performed using the SPM Anatomy toolbox (Eickhoff et al., 2005). To reveal the nature of the interactions, beta-values were extracted using the rfxplot toolbox (Glascher, 2009) implemented in SPM5. *t*-tests were performed over the extracted percentage signal change values to further investigate the functional properties of the areas of activation. Statistical analysis was performed with SPSS 14.0 software.

Finally, data were analyzed by using a second design matrix accounting for effects of the FRAME and the GAIN as factors, following the model previously used by some of us (Civai et al., 2012). We modeled the offers as a categorical factor with 3 levels fair (5€), middle (3€), and unfair (1€) yielding to a 2 × 3 factorial design with six conditions and their temporal derivative. The rest of the analysis was carried out as in the first model. With respect to the effect of frame, in this analysis we were interested at the contrast gain 5: “I take” vs. “I give you.” We reasoned that “gain 5€” could represent a good testing condition for investigating the frame effect, since it is an equal fair offer and the effect of frame on the perception of unfairness should be null; finding an effect of “I take” vs. “I give you” frames for gain 5 would strengthen the idea that the way offers are framed influence how people perform the task.

### Statistical analyses of behavioral data

SPSS for Windows (version 14.0) was used for performing a repeated measure ANOVA with within-subject factors type of “frame” (“I take,” “I give you”) and “gain” (1€, 3€, 5€) on the subjects' rejection rates and response times (RTs) data. All *post-hoc* comparisons between single factors were carried out using LSD Fisher's test (α ≤ 0.05).

## Results

### Behavioral results

At the debriefing, all participants reported that they believe the offers came from genuine humans.

#### Rejection rates

**Figure 1:**
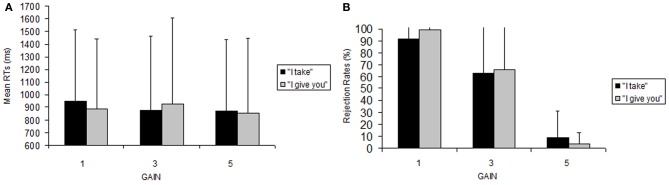
**Behavioral data**. Reaction time **(A)** and accuracy **(B)** data for performing the UG task. Error bars indicate standard error (SEM).

We found a significant main effect of gain, *F*_(2, 32)_ = 23.91, *p* < 0.001, with significantly less rejections for gain 5€ vs. 1€ (mean ± sem, 7.18 ± 4.43 vs. 75.8 ± 10.53, *p* < 0.001), and for 5€ vs. 3€ (7.18 ± 4.43 vs. 52.2 ± 11.65, *p* < 0.002) compared with 3€ vs. 1€ (52.2 ± 11.65 vs. 75.8 ± 10.53, *p* = 0.076, n.s.) (See Figure 1). The main effect of frame [*F*_(1, 16)_ = 0.35, *p* = 0.56] and the frame × gain interaction [*F*_(2, 32)_ = 0.8, *p* = 0.45] were not significant.

Identical effects were found also when we removed from the analysis four participants who never rejected an offer, because it was necessary that all the subjects considered had rejections in both the frames in order to perform an ANOVA without empty cells [frame, *F*_(1, 12)_ = 0.93, *p* = 0.76, n.s., η^2^ = 0.0022; gain, *F*_(2, 24)_ = 41.5 *p* < 0.001 η^2^ = 0.679, with significantly less rejections for gain 5€ vs. 1€ (5.98 ± 16.12 vs. 95.29 ± 15.36, *p* < 0.001), and for 5€ vs. 3€ (5.98 ± 16.12 vs. 64.42 ± 45.08, *p* < 0.002) compared with 3€ vs. 1€ (64.42 ± 45.08 vs. 95.29 ± 15.36, *p* = 0.070, n.s.); frame × gain, *F*_(2, 24)_ = 0.94, *p* = 0.40, n.s. η^2^ = 0.0201].

### fMRI results

#### Task-related network

**Figure 2:**
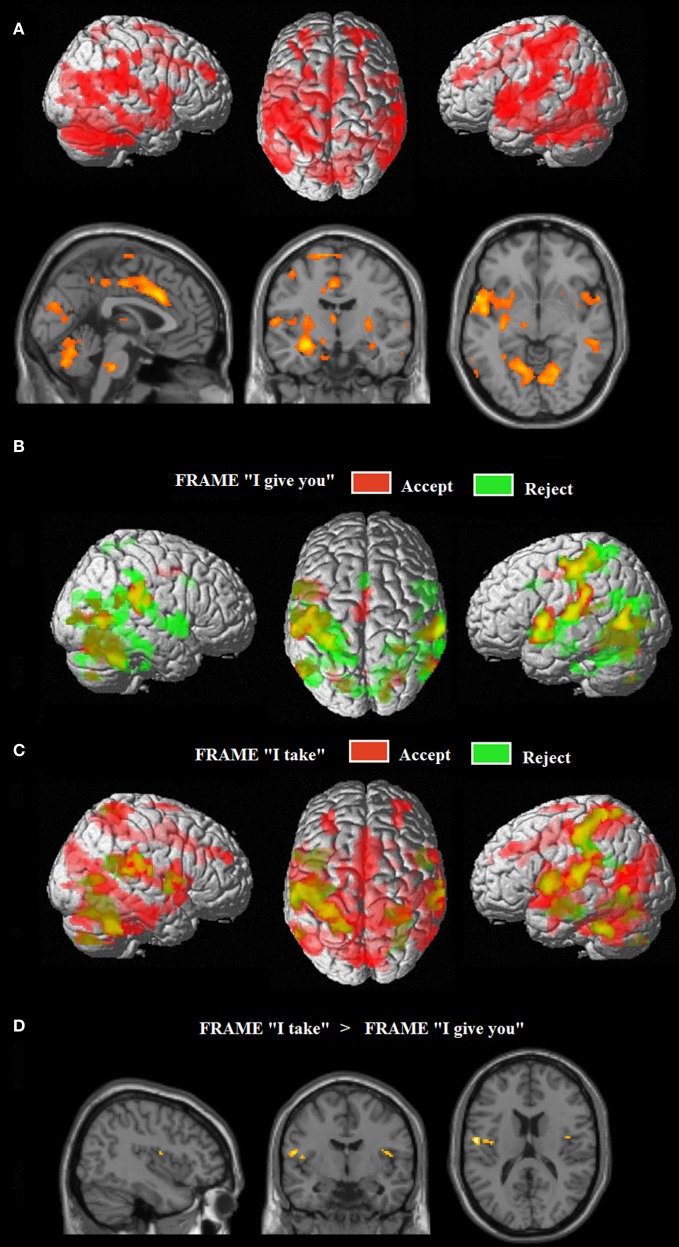
**(A)** Common neural networks associated with the UG task; network of areas differentially recruited by response “reject” in green (relative to “accept,” in red) for frame “I give you” **(B)** and for frame “I take” **(C)**. Activations are displayed on a rendered template brain provided by spm5. **(D)** Insula/rolandic opercular areas differential recruitment by the frame “I take” (relative to frame “I give you”) displayed on a single subject template brain provided by spm5.

The extensive network of areas recruited by the task (task > implicit baseline contrast) involved clusters of activity in: (i) the cerebellum bilaterally, extending to the inferior and to the superior temporal gyrus, the amygdala, the insula and the superior parietal lobe; (ii) the left middle temporo-occipital gyrus; (iii) the middle cingulate cortex, extending to the left supplementary motor area (SMA); (iv) the superior frontal gyrus bilaterally, extending to the right SMA; and (v) the middle frontal gyrus bilaterally (see Figure 2A, Table 1).

**Table 1:** **Whole brain analysis for the model accounting for *frame* and *type of response* related effects: brain regions showing significant relative increases of BOLD response associated with the experimental conditions**.

**Region**	**Side**	**MNI**			***Z***	**Cluster voxel**
		***x***	***y***	***z***		
**TASK-RELATED NETWORK**						
Cerebellum	R	30	−46	−34	5.65	22,735
Cerebellum	L	−14	−64	−28	5.40	
Insula	L	−42	12	2	4.97	
Insula	R	44	4	2	4.90	
Amygdala	L	−12	22	−16	4.77	
Amygdala	R	36	0	−14	4.71	
Inferior temporal gyrus	R	56	−30	−14	4.70	
Temporal pole	L	−50	10	−10	4.69	
Superior parietal cortex (Area 1)	L	−50	−20	50	4.68	
Superior temporal gyrus	L	−40	−26	16	4.65	
Inferior temporal gyrus	R	52	−26	−16	4.55	
Middle temporal gyrus	L	−52	−66	0	4.80	1187
Middle occipital gyrus	L	−54	−72	14	4.25	
Middle cingulate cortex	M	−2	16	34	4.50	1036
SMA	L	2	4	48	3.93	
Superior frontal gyrus	L	−22	16	56	4.42	121
Superior frontal gyrus	L	−12	−8	72	4.33	306
SMA	R	6	2	66	4.02	
Middle frontal gyrus	R	24	50	26	4.12	249
Superior frontal gyrus	R	14	56	24	3.94	
Middle frontal gyrus	L	−28	48	26	3.92	275
Middle frontal gyrus	R	26	26	38	3.58	108
Superior frontal gyrus	R	24	16	40	3.56	
**MAIN EFFECT OF TYPE OF FRAME: “I TAKE” > “I GIVE YOU”**						
Insula, Rolandic operculum	L	−55	−8	16	4.76^*^	41
Insula, Rolandic operculum	L	−38	−8	18	4.22^*^	45
Insula, Rolandic operculum	R	46	−6	16	3.63^*^	17
**(“I TAKE”_ACCEPT > “I TAKE”_REJECT) > (“I GIVE YOU”_ACCEPT > “I GIVE YOU”_REJECT)**						
Precuneus	R	10	−56	48	4.78	431
Superior parietal lobe (Area 7a)	R	38	−48	60	4.62	
Posterior insula, Rolandic operculum	R	44	−14	14	4.66	188
Calcarine gyrus	R	24	−60	6	4.07	260
Cuneus	R	16	−76	20	3.97	
Anterior cingulate	L	−4	18	22	4.25	59
Superior parietal lobe (Area 7a)	L	−20	−54	60	4.25	62
Superior parietal lobe (Area 2)	L	−22	−52	50	4.03	
Superior temporal gyrus	R	58	−6	4	4.03	63

For each region of activation, the coordinates in MNI space are given in reference to the maximally activated voxel within an area of activation, as indicated by the highest Z-value (p < 0.05, corrected for multiple comparisons at the cluster level, height threshold p < 0.001, uncorrected).

* pSVC < 0.05, corrected. L/R, left/right hemisphere.

#### Effects of the FRAME and RESPONSE TYPE (i.e., reject or accept)

Figure 2 show the fMRI results for the response-related effects: network of areas differentially recruited by response “reject” (relative to “accept”) for frame “I give you” **(B)** and for frame “I take.” **(C)** For the main effect of frame: “*I take” vs. “I give you” (and vice versa)*, no differential activation was found at the predefined statistical threshold. Based on previous neuroimaging studies on the framing effect described in the introduction (De Martino et al., 2006), we hypothesized that emotional areas such as anterior insula and amygdala, were involved in processing the frame; moreover, further imaging studies reported activations in the operculo/insular cortex associated with pain processing (e.g., Lötsch et al., 2012) and in interoceptive awareness and the representation of visceral responses associated with emotional situations (Lamm et al., 2007). Thus, a hypothesis-driven region of interest (ROI) analysis (Friston, 1997) was performed in which we tested significant increases of neural activity in the operculo/insular cortex [Anatomy Toolbox (Eickhoff et al., 2005)], for the main effect of frame. We found significant activation within the operculo/insular cortex bilaterally associated with the frame “I take” vs. “I give you.” No differential activation was found for the reverse comparisons (see Figure 2D, Table 1). In the ROI analysis performed on the amygdala [Anatomy Toolbox (Eickhoff et al., 2005)] for the main effect of frame we did not found significant activation within this area.

#### Frame × response type interaction: [(FRAME_I take: Accept>Reject)>(FRAME_I give you: Accept > Reject)] (and vice versa)

**Figure 3:**
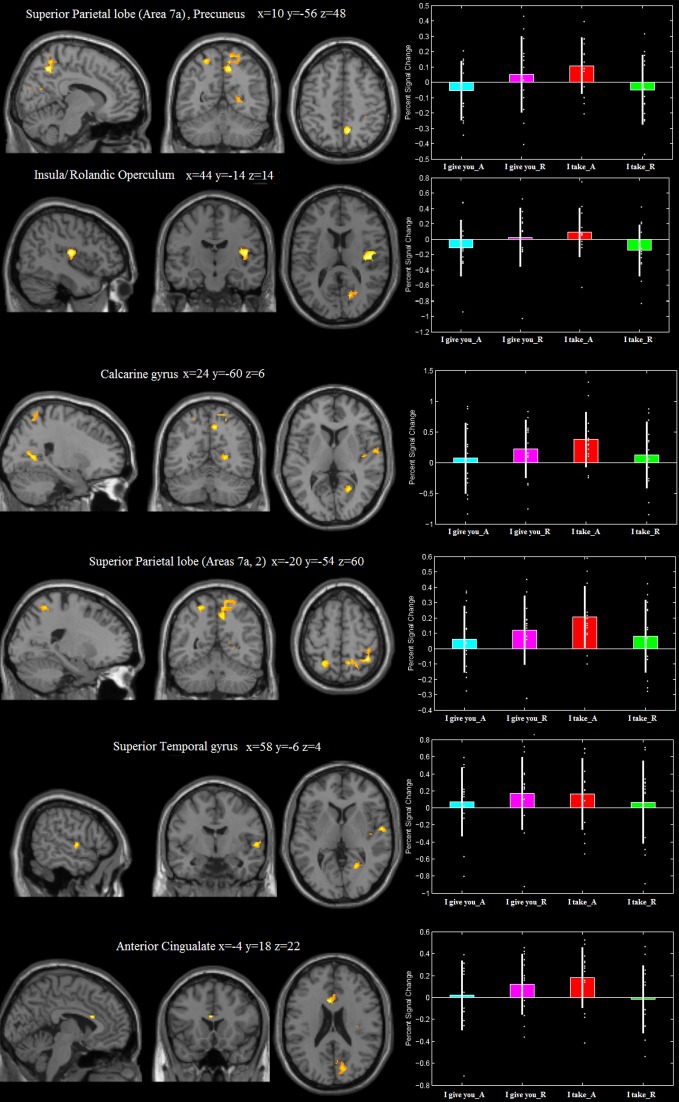
**Areas differentially recruited by the frame × type of response interaction [(“I take”_Accept > “I take”_Reject) > (“I give you”_Accept > “I give you”_Reject)]**. Group mean beta-values extracted from each of the activation clusters. The plots were created by using rfxplot [http://rfxplot.sourceforge.net/, (Glascher, 2009)].

The frame “I take” for accepted trials (vs. rejected) controlled for the frame “I give you,” differentially activated (i) the right precuneus, extending to the right superior parietal lobe (Area 7a), (ii) the right rolandic operculum/insular cortex, (iii) the right calcarine gyrus, extending to the right cuneus, (iv) the right superior temporal gyrus, (v) the left superior parietal lobe (Area 7a) extending to the left Area 2, and (vi) the left anterior cingulate (see Figure 3, Table 1). This interaction was due to an increase of neural activity for accepting frame “I take” vs. rejecting (*p* = 0.008, *p* = 0.022, *p* = 0.001, *p* = 0.023, *p* = 0.011, *p* = 0.04, respectively), which was significantly higher than that associated with accepting frame “I give you” vs. rejecting (*p* = 0.09, *p* = 0.08, *p* = 0.09, *p* = 0.1, *p* = 0.1, *p* = 0.08, n.s., respectively). No differential activation was found at the predefined statistical threshold for the reverse comparisons.

#### Gain-related effects

This model accounted for effects of the FRAME (i.e., “I take,” “I give you”) and the GAIN [i.e., fair (5€) middle (3€) and unfair (1€)] factors. Irrespective of frame, unfair (1€) gain [vs. middle gain (3€)], differentially activated the anterior cingulate cortex (ACC). Furthermore, fair (5€) gain [vs. middle gain (3€)], differentially activated (i) the left superior parietal lobe (Area 7a), extending to the precuneus, (ii) the middle cingulate cortex.

**Figure 4:**
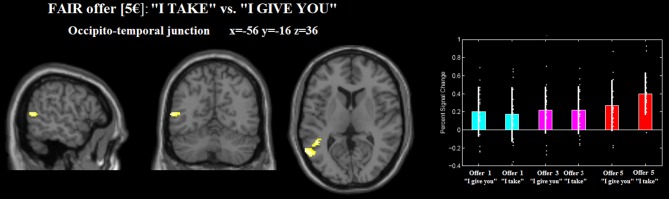
**Occipito-temporal junction differentially recruited by gain 5€ _“I take” (relative to 5€ _“I give you”)**. The plot were created by using rfxplot [http://rfxplot.sourceforge.net/, (Glascher, 2009)].

For the fair (5€) gain, the frame “I take” (vs. “I give you”) differentially activated the left occipito-temporal junction and the middle temporal gyrus (see Figure 4, Table 2). No differential activation was found at the predefined statistical threshold for the other comparisons (see Table 2).

**Table 2:** **Whole brain analysis for the model accounting for *frame* and for *gain* effects: brain regions showing significant relative increases of BOLD response associated with the experimental conditions**.

**Region**	**Side**	**MNI**			**Z**	**Cluster voxel**
		***x***	***y***	***z***		
**MAIN EFFECT OF FRAME: “I TAKE” vs. “I GIVE YOU”**						
–	–	–	–	–	–	–
**MAIN EFFECT OF FRAME: “I GIVE YOU” vs. “I TAKE”**						
–	–	–	–	–	–	–
**MAIN EFFECT OF GAIN: 1€ > 3€**						
Anterior cingulate cortex	R	18	4	32	6.51	150
**MAIN EFFECT OF GAIN: 3€ > 1€**						
–	–	–	–	–	–	–
**MAIN EFFECT OF GAIN: 1€ > 5€**						
–	–	–	–	–	–	–
**MAIN EFFECT OF GAIN: 5€ > 1€**						
–	–	–	–	–	–	–
**MAIN EFFECT OF GAIN: 3€ > 5€**						
–	–	–	–	–	–	–
**MAIN EFFECT OF GAIN: 5€ > 3€**						
Superior parietal lobe (Area 7a)	L	−12	−60	58	4.49	619
Precuneus	L	−4	−58	54	3.92	
Middle cingulate cortex	M	−4	0	40	4.11	125
**GAIN 5: “I TAKE” > “I GIVE YOU”**						
Occipito-temporal junction	L	−56	−62	8	3.84	159
Middle temporal gyrus	L	−62	−56	8	3.81	
**GAIN 5: “I GIVE YOU” > “I TAKE”**						
–	–	–	–	–	–	–
**GAIN 1: “I GIVE YOU” > “I TAKE”**						
–	–	–	–	–	–	–
**GAIN 1: “I GIVE YOU” > “I TAKE”**						
–	–	–	–	–	–	–
**GAIN 3: “I GIVE YOU” > “I TAKE”**						
–	–	–	–	–	–	–
**GAIN 3: “I GIVE YOU” > “I TAKE”**						
–	–	–	–	–	–	–

For each region of activation, the coordinates in MNI space are given in reference to the maximally activated voxel within an area of activation, as indicated by the highest Z-value (p < 0.05, corrected for multiple comparisons at the cluster level, height threshold p < 0.001, uncorrected). L/R, left/right hemisphere. As all the values are value p < 0.05, corrected for multiple comparisons at the cluster level, height threshold p < 0.001.

We performed also an analysis by including a parametric modulator for the factor “gain.” The parametric approach did not yield significant results at the predefined threshold.

## Discussion

In this fMRI study we have investigated the neural mechanisms underlying the economical decisions people make when the information on which they rely is formulated in terms of gain or loss. Participants played a modified fMRI version of the UG with different bids preceded by two different frames: the expression “I give you” (*gain*) focusing on money the respondent would receive if she/he agreed with the proponent, and the expression “I take” (*loss*) focusing on the money that would be removed from the respondent in the event that she/he accepted the offer. Behaviorally, unfair offers were equally often rejected in both conditions. This is different from what was found in the study by Sarlo et al. (2012) in which participants rejected more when the offer was framed as a loss rather than as a gain. Our failure to confirm this result may be due to the smaller sample of subjects employed in the present study.

### Task-related network

Since the task we used was a modified version (Sarlo et al., 2012) of the classical UG, we first describe the task-related network. Overall, the network that was associated with the task included clusters of activation in key areas which have been classically found in previous studies investigating the neural underpinning of the UG (e.g., Sanfey et al., 2003; van't Wout et al., 2005; Knoch et al., 2006, 2008; Koenigs and Tranel, 2007; Tabibnia et al., 2008; Moretti et al., 2009; Güroðlu et al., 2010, 2011; Civai et al., 2012; Corradi-Dell'Acqua et al., 2012), such as the insular cortex bilaterally, the middle cingulate cortex, the superior frontal and the middle frontal gyri bilaterally, the inferior and superior temporal lobe. Interestingly, the task-related network included also the amygdala bilaterally, known to be related to the mediation of aggressive responses (Nelson and Trainor, 2007) and of biasing decision-making (Bechara et al., 2003; De Martino et al., 2006), and it has been found activated also in a previous fMRI study on the UG (Gospic et al., 2011).

Structures normally involved in mental calculation (Rickard et al., 2000; Zago et al., 2001; Hanakawa et al., 2003), such as the right parietal and the right precuneus, were significantly activated in the frame-by-decision interaction, in which the responder takes but, in this condition, the player accepts. When one accepts in the loss frame, one deviates more from her “expected” response. This deviation from the behavior we expect from her, could be accompanied by an increase of mental calculation resources and processing.

Importantly, all these activations have been always related to processes such as emotional processing (Sanfey et al., 2003), theory of mind (Gallagher and Frith, 2003; Amodio and Frith, 2006), cognitive processing (Sanfey et al., 2003) such as executive control, goal maintenance, and the monitoring/control of one's emotional responses (van't Wout et al., 2005; Knoch et al., 2006, 2008; Koenigs and Tranel, 2007; Moretti et al., 2009; Güroðlu et al., 2010, 2011; Baumgartner et al., 2011) triggered by the task.

### Frame by decision interaction in the operculo-insular cortex and the anterior cingulate cortex

Despite the lack of significant interaction at the behavioral level, at the neural level we observed a frame-by-response interaction, revealing an increase of neural activity in the right rolandic operculum/insular cortex. This interaction was due to an increase of neural activity for accepting frame “I take” vs. rejecting, which was significantly higher than that associated with accepting frame “I give you” vs. rejecting. Interestingly a hypothesis-driven ROI analysis performed for testing significant increases of neural activity in the operculo/insular cortex, showed a significant activation within the operculo/insular cortex bilaterally associated with the frame “I take” vs. “I give you.” Sanfey et al. (2003) found that a stronger activation in the anterior part of the insula when evaluating an unfair offer was associated to rejections. In contrast, in our study we found a stronger activation in the posterior part of the insula for accepting (compared with rejecting) the frame “I take” vs. “I give you.” We interpreted the acceptance effect we found as related to a discrepancy between expected response and my decision [see also Güroðlu et al. (2010), for a similar interpretation]. Our results extend the interpretation of the role of the insula put forward by Sanfey et al. (2003) and suggest that this region may be characterized by two different functions: the anterior part of the insula might evaluate the outcome, while the posterior part of the insula might evaluate the response to the outcome. Based on our findings we can add that the posterior insula is also sensitive to the frame in which offers have been formulated.

Our cluster of activation in the operculo-insular cortex is localized more posterior than the usual one found in the anterior insula in UG fMRI studies, e.g., Sanfey et al. (2003) and also the one found in previous studies performed by some of us (Civai et al., 2012; Corradi-Dell'Acqua et al., 2012). Nevertheless, it has been shown that among other regions, such as the thalamus, the insular, anterior cingulate, primary and secondary somatosensory, premotor and supplementary motor cortices, the operculo-insular cortex is a crucial part of the pain matrix (Treede et al., 1999; Peyron et al., 2000; Apkarian et al., 2005; Bingel and Tracey, 2008). This is particularly relevant since the activation found in the anterior insula during the UG have been classically interpreted as unfair offers triggering negative emotions, given that many studies have found a crucial involvement of this area in processing emotional states, pain and distress (Damasio et al., 2000; Calder et al., 2001; Wicker et al., 2003; Corradi-Dell'Acqua et al., 2011). Evidence for the operculo-insular cortex involvement in pain processing came from studies using PET, evoked potentials or fMRI techniques (Peyron et al., 2002; Frot et al., 2007; Baumgartner et al., 2010; Oertel et al., 2012), and from studies involving direct stimulation of this area (Mazzola et al., 2009) and of the insular cortex (Mazzola et al., 2006). Other authors have previously found activation in a cluster localized more posterior than the anterior insula. In one of those studies, the authors (Wright et al., 2011) used a modified version of the UG and varied the social context, by inducing thus a bias in participants acceptance of objectively identical offers. They found that the objective social inequality was integrated with social context in posterior and mid-insula. Consistently, in another study (Hsu et al., 2008) it has been shown that posterior insula activity negatively correlated with inequality.

The frame × response type interaction contrast included also the fair €5 offers. We ruled out the possibility that the fair €5 offers drove the effect, since the rejection rates for fair €5 offers in the “I take” frame did not significantly differ from the frame “I give.” We also would like to argue that gain 5€, being the most equal gain, is the condition that more than the others shows the frame effect: precisely because it is an equal and fair offer, the effect of frame should be null. Instead, we found that the activation in the OT junction was significantly modulated by the effect of frame for fair, €5 offers.

In our study the activation of the operculo-insular cortex was significantly increased when participants accepted (vs. rejected) the offers presented in the frame “I take,” as compared to the frame “I give you.” We reasoned that in the loss frame one should be more prone to reject with respect to the gain frame; it follows that when participants accept in the loss frame they deviate more from their “expected” response, even though this interpretation is speculative, as we cannot provide behavioral evidence to support the expectancy hypothesis. Accordingly, the operculo-insular cortex might signal this deviation from participants' own expected behavior. It has been proposed that the equal treatment is a default social norm, and its violation is signaled by the anterior insula (Civai et al., 2012). Further evidence supporting the view that the anterior insula signals the level of inequity aversion, and, more broadly, norm violations came also from another fMRI study (Hsu et al., 2008) in agreement with the idea that anterior insula plays a critical role in detecting social norm violations (Spitzer et al., 2007; King-Casas et al., 2008; Strobel et al., 2011), thus extending its role beyond emotional involvement (Sanfey et al., 2003). Importantly, it has been shown that the frame “I take,” by acting as a loss frame, elicited the characteristic defensive response pattern that is evoked by aversive stimulation, in which increases in skin conductance are coupled with increases in heart rate (Güth et al., 1982; Sarlo et al., 2012). To sum up, the role of the anterior insula in the UG in the studies reviewed above is comparable with the one we found in the operculo-insular cortex. In addition, we add that the operculo-insular cortex is modulated by the frame in which the offers are formulated.

A further interpretation might be that operculo-insular cortex activation could be somewhat related to processes of agency-attribution and/or adoption of an egocentric vs. allocentric reference frame, and the effect may arise from the “linguistic” context involving the proposer alone (“I take”) or the proposer along with the responder (“I give you”), thus modulating the activity of mechanisms of self-other distinction that are associated with posterior insula and rolandic operculum (see Vogeley and Fink, 2003; Sperduti et al., 2011). In our study, the activation of the operculo-insular cortex was significantly increased when participants were processing the frame “I take,” as compared to the frame “I give you” and accepted (vs. rejected) the offers. Here agency has to be attributed to the person to whom the proposal of how to split the money is made, independent of the frame “give” or “take.” This might appear in contrast with the role played by the insula in first person perspective attribution (see Vogeley and Fink, 2003; Sperduti et al., 2011) unless the player imagine changing his own perspective in to the proposer's one.

Consistently with previous studies in which the ACC showed an increased activation for unfair compared with fair offers, e.g., Sanfey et al. (2003), we found an increased activation in the ACC for unfair 1€ as compared to mid-value 3€ gains. Some authors, e.g., Sanfey et al. (2003) argued that the ACC has been implicated in detection of cognitive conflict (Botvinick et al., 1999; MacDonald et al., 2000), and the activation of the ACC in the context of the UG is related to the conflict between cognitive and emotional motivations. As a new feature, we also found that the ACC activation in the frame by response interaction, with increased activation for accepting (vs. rejecting) gains presented in the frame “I take,” as compared to those presented in the frame “I give you,” which corresponds to the most unfair condition, albeit participants accept that the proposer takes money. This condition might trigger a conflict between cognitive and emotional motivations, which in turns activates the ACC. It has been suggested that together with the insula, the ACC activation might be related to behavior that deviates from participants' personal standards (Güroðlu et al., 2011). It has been shown that, by varying degrees of intentionality, the ACC activation was increased for accepting unfair offers in the no-alternative context and for rejecting an unfair offer in fair- and hyperfair-alternative contexts (Güroðlu et al., 2011). Taken together, these results indicate that accepting that the proposer takes the money, independent of the gain, is indeed a deviant choice with respect to what one normally does (Güroðlu et al., 2011).

### Implicit mental simulation mechanisms triggered by the UG

That the UG could trigger mechanisms related to mental simulation has never been proposed. With the term simulation we refer to the mental process by which people mentally visualize, or move or feel and experience situations, which occurs in the absence of the appropriate external stimuli or sensory input (mental imagery is sometimes colloquially referred to as “visualizing,” “seeing in the mind's eye,” “hearing in the head,” “imagining the feel of,” etc.) (Kosslyn et al., 1995a, 2001). It has been largely accepted that people use mental imagery, for instance, during memory retrieval, problem solving, producing descriptions, mental practice, and motivational states (Kosslyn, 1980). Thus, a mental process involving a first or third person perspective could well be carried out through imagery (Vogeley et al., 2004). Importantly, mental imagery can occur after explicit instructions (Jeannerod, 1999) but it can also be implicitly triggered (Jeannerod and Frak, 1999); implicit mental imagery occurs when subjects, even if they receive no instruction to imagine, unconsciously imagine the scene or the action while performing another task, e.g., during mental rotation of body parts (e.g., Zacks et al., 1999; Kosslyn et al., 2001), handedness recognition of a visually presented hand (e.g., Parsons and Fox, 1998), judgment as to whether an action would be easy, difficult or impossible (Johnson et al., 2002), or recognizing and understanding actions of other individuals (e.g., Johnson et al., 2002). In performing the UG, although subjects received no instruction to do so, they could represent in their mind of the imagine the action associated with the task. That individuals imagine the situations as if they were real and feel pain when the most disadvantageous conditions are encountered could well explain why regions found associated in processing pain such as the opercular/insular cortex were found activated when subjects performed the UG. It is conceivable that while performing the UG the participants (implicitly) simulate sensations, actions, emotions, anticipating the action consequences, switch between first and third person perspective, although not instructed to do so. Accordingly, in our study we found significant clusters of activation in areas involved in mental imagery, strongly suggesting that one of the mechanisms supporting the UG performance could well be mental imagery. Indeed, at variance with the results previously found in fMRI studies on the UG, interestingly the task-related network included also a significant activation in the left superior parietal cortex, which was localized in the primary somatosensory area (Area 1). This activation is typically found in studies in which subjects actually experience the sensation or the movement, or when they imaging them (Tomasino et al., 2010). This finding may be interpreted as if the subjects implicitly simulated the gain/loss. Somatoperception corresponds to the process of perceiving the body itself, and particularly of ensuring somatic perceptual constancy (Longo et al., 2010). The somatosensory cortex is reported in studies requiring mapping of subjective feeling states arising from bodily responses (Critchley et al., 2004). It is relevant here the role of somatosensory cortices in sensory imagery of affectively-significant states. Somatosensory-based memories can be reactivated by the anterior emotion network (Damasio, 1994). It has been shown that repetitive transcranial magnetic stimulation (rTMS) to the face S1 representation impaired recognition of facial emotional expressions (Pitcher et al., 2008) and that the observation of erotic images or mutilated bodies as compared to neutral items activated the right SI and SII (Rudrauf et al., 2009). The S1 activation during the UG task thus might be related both to an increased attention to one's bodily states as if the neural representation of the experiencing subject's body is a vehicle of their emotional experience (Longo et al., 2010). The UG is a self-centered task, thus it is reasonable that the left S1 and area 2 activations might reflect mental imagery of the sensations they would physically experienced during the UG.

With respect to the parietal lobe, we found that the left superior parietal lobe (Area 7a) was significantly activated independent of the frame in which offers were formulated, i.e., “I give you” or “I take” by gain 5€ as compared to 3€, thus for equal offers. In addition, the left area 7a was significantly activated by the frame by decision interaction, in which the responder takes but this time the player accepts. When you accept in the loss frame you deviate more from your “expected” response. It is not only the insula signaling this deviation from your own expected behavior, but also area 7, which has been related to egocentric (body- and body part-centered) coordinates coding (Makin et al., 2007), to the processing of multimodal integrated spatial representations in body-centered coordinates (Felician et al., 2004) and to updating postural representations of the upper limb (Pellijeff et al., 2006). In a previous study, some of us found that the left posterior IPS codes an allocentric, but not egocentric, visual model of the body (the body structural description) (Corradi-Dell'Acqua et al., 2009). Taken together, these studies suggest that a left area 7a activation may reflect a continuous updating of egocentric and allocentric coordinates while playing the UG. Another sub-region of the left parietal lobe we found activated in the frame by decision interaction was area 2, which is a somatosensory area, e.g., Grefkes and Fink (2005). The cluster of activation in area 7a extended including also the left precuneus. This region has been found activated in studies addressing episodic memory and the creation of imaginary or future personal scenarios (Cavanna and Trimble, 2006; Buckner and Carroll, 2007). It is possible that participants implicitly imagine the offers in terms of past or hypothetical future scenarios and fictive losses, see Kirk et al. (2011), or they could implicitly simulated situations in which the responder takes and the player accepts shifting from a 1st or a 3rd person perspective imagery (Ruby and Decety, 2001).

We also found that the left occipito-temporal junction was activated for the equal offer (“I take” vs. “I give you” for gain 5€). Our coordinates of the left occipito-temporal junction cluster are in the proximity to previously reported locations of extrastriate body area (EBA) in human brain (Arzy et al., 2006; Corradi-Dell'Acqua et al., 2009). Previous studies implicated the EBA by many body-part related processes including self-generated (Astafiev et al., 2004) and goal-directed (Takahashi et al., 2008) movements, as well as reaching to kinesthetically defined targets (Darling et al., 2007) and during imagery of the tool-use in near and far space (Tomasino et al., 2012). In this vein, the occipito-temporal activation clusters are modulated by the equal offer 5€, which can be hypothesized as being more unpleasant by the frame “I take” where is the proposer taking the money. Accordingly, the activation could be related to the generation of an action that might be considered a social confrontation, such a rejection. In the same line, it has been shown that activation of the middle occipito-temporal cortex was modulated by emotional and social information while participants viewed and categorized affective pictures that varied on two dimensions: emotional content (i.e., neutral, emotional) and social content (i.e., faces/people, objects/scenes) (Norris et al., 2004).

We found that the right calcarine gyrus and the right cuneus were significantly activated by the frame by decision interaction, in which the responder takes but this time the player accepts. We can exclude that this activation is related to visual processing of the stimuli since it is a product of the interaction term. Rather, we suggest that in this condition there is an increase of implicit visual imaginary processes, as described above, which triggers an increase of activation in areas related to visual imagery of scenes and characters (Kosslyn et al., 1995a,b, 2001; Kosslyn and Thompson, 2003), and an increase of activation in areas related to episodic memory retrieval during imagery such as the cuneus (Fletcher et al., 1995). It has been shown that V1 can be activated whenever images are formed, even if they are not necessarily used to perform a task (Klein et al., 2000). In that study, authors (Klein et al., 2000) used event-related fMRI to detect and characterize the activity in the calcarine sulcus during mental imagery. The results revealed reproducible transient activity in this area whenever participants generated or evaluated a mental image. This transient activity was strongly enhanced when participants evaluated characteristics of objects, whether or not details actually needed to be extracted from the image to perform the task (Klein et al., 2000). Interestingly, it has been shown that functional activation was larger in the right than the left hemisphere and larger in the occipital than in the occipitoparietal regions during processing of a series of pleasant, neutral, or unpleasant pictures (Lang et al., 1998). Importantly, both emotional and neutral pictures produced activity centered on the calcarine fissure, only emotional pictures also produced sizable clusters bilaterally in the occipital gyrus and in the right fusiform gyrus (Lang et al., 1998), and we argue probably related to implicit imagery processes.

To conclude, we argue that areas involved in imaginary task were found activated by our version of the UG, mainly in the frame by decision interaction, in which the responder takes and the player accepts. This condition corresponds to a loss frame, and here participants deviate from their “expected” response. Thus, deviation from participants' own expected behavior are signaled not only by the posterior insula/rolandic operculum but also trigger an increase of activation in areas related to mental imagery. Our findings extend the current understanding of the neural substrate of social decision making, by disentangling the structures sensitive to the way in which the information is formulated, i.e., framing effect, in terms of gain or loss, which influences people's decisions.

### Conflict of interest statement

The authors declare that the research was conducted in the absence of any commercial or financial relationships that could be construed as a potential conflict of interest.

## Appendix

### Instructions

During the experiment you will be asked to take part to the present scenario.

Another participant has been given the amount of 10 euro and that he/she has to decide how to split the amount of 10 euro with you. You cannot negotiate the proposal. You have the possibility to accept or reject the proposal, considering that:

- if you accept the offer, both (you and the proposer) will get the money as suggested;
- if you reject the offer, none of you would get any of the money.

Our study aim at observing the behavior of both the proposers, i.e., who proposes how to split the amount of money, and of the responder, who are asked to decide whether accepting or rejecting the offers.

In the past month we have involved (and scanned) a number of participants, asked to decide how they would split the amount of 10 euros, considering the rules mentioned above. The offers that you will be presented with, are therefore the proposals made by these participants.

All the participants, both the proposers and those who participated as responders like you, will receive a fixed compensation for participating in the experiment corresponding to 15 euro, plus a compensation equal to 10% of the amount of money gained.

You will not be able to identify the offers made by the single previous participants since the different proposals will be presented in a random order. The program will allow the experimenter only to load later on the offers made by the single proposers so to assign them the respective compensation.
